# The functional role and diversity of soil nematodes are stronger at high elevation in the lesser Himalayan Mountain ranges

**DOI:** 10.1002/ece3.8061

**Published:** 2021-09-24

**Authors:** Yasmeen Kouser, Ali Asghar Shah, Sergio Rasmann

**Affiliations:** ^1^ Department of Zoology Nematode Biodiversity and Genomics Research Lab Baba Ghulam Shah Badshah University Rajouri India; ^2^ Laboratory of Functional Ecology Institute of Biology University of Neuchâtel Neuchâtel Switzerland

**Keywords:** Alpine environment, elevation gradient, function trophic structure, metabolic footprint, soil biodiversity, soil ecology

## Abstract

Soil nematodes are a foremost component of terrestrial biodiversity; they display a whole gamut of trophic guilds and life strategies, and by their activity, affect major ecosystem process, such as organic matter degradation and carbon cycling. Based on nematodes' functional types, nematode community indices have been developed, and can be used to link variation in nematodes community composition and ecosystem processes. Yet, the use of these indices has been mainly restricted to anthropogenic stresses. In this study, we propose to expand the use of nematodes' derived ecological indices to link soil and climate properties with soil food webs, and ecosystem processes that all vary along steep elevation gradients. For this purpose, we explored how elevation affects the trophic and functional diversity of nematode communities sampled every 300 m, from about 1,000 m to 3,700 m above sea level, across four transects in the lesser Himalayan range of Jammu and Kashmir. We found that (a) the trophic and functional diversity of nematodes increases with elevation; (b) differences in nematodes communities generate habitat‐specific functional diversity; (c) the maturity index (ΣMI) increases with elevation, while the enrichment index decreases, indicating less mature and less productive ecosystems, enhanced fungal‐based energy flow, and a predominant role of nematodes in generating carbon influxes at high‐elevation sites. We thus confirm that the functional contribution of soil nematodes to belowground ecosystem processes, including carbon and energy flow, is stronger at high elevation. Overall, this study highlights the central importance of nematodes in sustaining soil ecosystems and brings insights into their functional role, particularly in alpine and arctic soils.

## Graphical abstract

Soil nematodes trophic and functional diversity increases with elevation. Therefore, soil nematodes in alpine and arctic soil contribute mostly to ecosystem functioning.

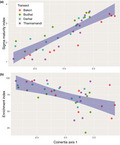

## 1 INTRODUCTION

It has been estimated that soils of terrestrial ecosystems sustain about 25% of the world biodiversity (Bach et al., 2020; Decaëns, 2008; Decaëns et al., 2006); consequently, soils function as biodiversity reservoirs, and have the potential to mainly contribute to ecosystem functioning (Bardgett & van der Putten, 2014; Decaëns, 2010; Fitter et al., 2005). Indeed, soil fauna functional diversity has been shown to contribute to ecosystem functioning by impacting on different processes, such as primary production and nutrient cycling of carbon, phosphorous, or nitrogen (Brussaard, 1997), the decomposition of organic matter, or the assimilation of carbon in food webs, which in turn regulates energy movements between the below and the aboveground compartments of the ecosystems (Hunt & Wall, 2002; Krumins et al., 2013).

The group of roundworms (i.e., the nematodes; phylum Nematoda) represents a major component of the belowground fauna diversity. Nematodes include more than 27,000 described species (Hodda, 2011; Hugot et al., 2001), are found almost in every inhabitable place on Earth, and represent about 80% of belowground bulk metazoan taxonomic and functional diversity (Bongers & Bongers, 1998; Hodda et al., 2009). Nematodes can be assigned to practically all existing trophic groups, including the herbivore, fungivore, bacterivore, predator, unicellular eukaryote feeder, parasite, and omnivore trophic and functional group (Yeates et al., 1993). Nematodes can also be functionally assigned to a wide gamut of ecological adaptations, ranging from being classified as “colonizer” (i.e., r strategists,) to being classified as “persister” (i.e., K strategists), and all in between, such as along the colonizer‐persister (“cp”) scale as described by Bongers (1990). Nematodes therefore constitute a key component of the soil microbiota, and contribute to regulating several ecosystem processes, such as mineral cycling, succession processes, and energy flow (Andrén et al., 1995; Bongers & Bongers, 1998; Boström & Sohlenius, 1986).

Numerous studies have demonstrated the critical role of climate in the development and maintenance of soil nematode diversity (Chen et al., 2015; Nielsen et al., 2014; Song et al., 2017). For instance, Nielsen et al. (2014) showed that nematode community composition was strongly related to two main climatic factors, mean annual rainfall and temperature, which accounted for 65% and 58% of the total variation in community differences, respectively. Similarly, mean annual precipitation has been shown to influence nematode assemblage at the regional scale (Chen et al., 2015). In addition, climate can directly impact on local soil and vegetation characteristics (Rodriguez‐Iturbe et al., 1999), and thus climate, indirectly, can influence soil invertebrate communities via changes in vegetation and soil properties (Kergunteuil, 2016). Therefore, contemporary and historical climatic factors can be used to study changes in species and functional diversity of soil nematodes across large geographic scales (Li et al., 2020).

In addition to climate, it is well established that soil nematode diversity, abundance, and composition are also influenced by soil physicochemical properties, such as soil temperature (de Ruiter et al., 1998), relative humidity (Dinoor & Eshed, 2003), organic matter content (Collins et al., 1995; Cook et al., 1992; Crawford et al., 2005; De Deyn et al., 2003, 2004), phosphorus (De La Peña et al., 2006), texture, or salinity (Djigal et al., 2004), either from the local to the large scales (Chen et al., 2015; Liu et al., 2016; Quist et al., 2019; van den Hoogen et al., 2019). As a consequence, the study of the taxonomic and functional structure of nematode communities can, in turn, be used for assessing soil quality (Brinkman et al., 2008; Sochová et al., 2006; Wilson & Kakouli‐Duarte, 2009), as well as for evaluating natural changes in soil ecological conditions, for instance, along large‐scale ecological and climatic gradients (Kergunteuil, 2016). Accordingly, several indices have been developed that summarize the functional role and the contribution of nematodes in the ecosystem (Bongers & Ferris, 1999; Ferris, 2010; Ferris et al., 2001). For instance, the “Channel index,” the “Enrichment index,” and the “Structure index,” which are all derived from calculating the weighted functional diversity components of the soil nematodes communities (Berkelmans et al., 2003; Ferris et al., 2001), represent the predominant decomposition pathways, food web response to available resources and state of food web affected by environmental stress, respectively (Ferris & Bongers, 2009; Ferris et al., 2001) Moreover, the “Metabolic Footprint,” which quantifies the amplitude of C utilization by different components of the nematode soil food web, can function as an indicator of carbon and energy flow in the soil (Ferris, 2010). Being integrators of ecosystems properties, we therefore expect these indices to vary across habitat types, as well as local climatic and edaphic conditions.

While studies relating nematodes' functional structure and soil functioning remain mostly restricted to anthropogenic systems (Freckman & Ettema, 1993; Šalamún et al., 2014; Zhao et al., 2015), studying functional variation of soil nematode communities in natural systems can inform on the potential natural relationship between belowground diversity, ecosystem function, soil properties, and climate (van den Hoogen et al., 2019). In this context, we here propose to expand the use of nematodes' derived ecological indices to study natural populations' variation along large‐scale ecological gradients, which in turn, will allow increasing our understanding of how soil nematodes contribute and inform on the changes in ecosystem functioning across contrasted landscapes (Ritz & Trudgill, 1999; Wilschut et al., 2019; Yeates, 2003). In this regard, studying nematode communities' functional variation along steep elevation gradients can be used to dissect the link between climate and soil conditions and nematode functional properties within a homogenous biogeographical and evolutionary background (Kergunteuil, 2016; Körner, 2007).

The purpose of this study was thus to investigate whether along elevation gradients, nematode communities and functionalities vary predictably with soil and climatic properties. For this, we studied soil community's composition along four elevation transects of Northern India (Jammu and Kashmir region). Based on previous studies along elevation gradients in the Alps (Kergunteuil, 2016), we hypothesized that (a) nematodes' functional composition varies with elevation, (b) nematodes' functional beta‐diversity covary with changes in soil and climatic conditions along the elevational gradient, and (c) indices related to ecosystem properties also vary with nematode communities being more associated with more productive and mature ecosystems at low versus high elevation. Interestingly, it was previously shown that along the Alpine elevation gradients, several soil nematodes' trophic and functional groups, such as the herbivores, increase with elevation (Kergunteuil, 2016). These findings were to some extent in opposition to classic hypotheses of biodiversity changes along elevation, in which, for most clades, theory predicts a decline in biodiversity with elevation, indeed due to an increase of more constrained and stressful environmental conditions at high elevation. By studying similar ecosystem dynamics but in an entirely different setting—the Himalayas versus the Alps—we ultimately hope to draw broader conclusions about soil nematodes' biodiversity patterns and soil functioning in nature.

## 2 MATERIALS AND METHODS

### 2.1 Study area

**FIGURE 1:**
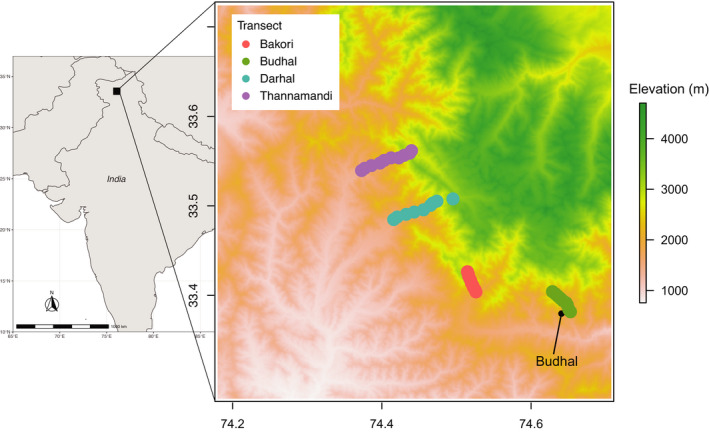
Sampling sites along elevation gradients. Shown is an elevation map of the Indian Jammu and Kashmir region in which four elevation transects were chosen for sampling nematode biodiversity

We surveyed soil nematode communities along the Pir Panjal mountain range, a group of mountains in the Lesser Himalayan region, running from east‐southeast to west‐northwest, and including the Indian Territory of Jammu and Kashmir, where this study was conducted, and where the average elevation varies from 1,000 m above sea level (a.s.l.) to 4,000 m a.s.l. Within this region, four elevation transects were selected viz., Darhal, Thanamandi, Budhal, and Bakori transects (Figure 1, Table S1). The transects span elevations ranging as low as about 1,000 m a.s.l., which are characterized by evergreen forests dominated by arboreal plant species, such as *Quercus leucotricophora, Pinus wallichiana, Pyrus pashia, Rhododendron arboreum,* and *Priensepia utilis*, and to almost 3,700 m a.s.l., habitats which are characterized by alpine meadows and recent glacial retreats (Table S1).

### 2.2 Extraction of climatic variables

To characterize the climatic conditions present at each site along the elevational transects, we extracted the 19 BIOCLIM variables from the Chelsa global climate dataset (https://chelsa‐climate.org/bioclim/) at 30‐s resolution (Karger et al., 2017). For statistical analyses, we removed overly correlated variables from the full list using the package *caret* (Kuhn et al., 2020), and ended up with 10 variables describing the climatic niche of each site (Table S2).

### 2.3 Soil and nematode sampling

Nematodes were sampled between June and October 2020 across the four transects, and starting end of June at the lowest sites, and finishing in October at the highest elevation sites. Within each transect, we sampled 10 sites, separated from one another about 300 m in elevational distance so as to yield a total of 40 sites (Table S1). At each site, a sampling quadrat of 2 × 2 m was randomly chosen within characteristic and homogenous vegetation type of the site. In sub‐alpine areas, soil samples were predominantly collected within *Fagus sylvatica, Abies pindrow, Pinus* spp., *Quercus* spp., or *Castanea sativa‐*dominated forests, whereas sampling in the alpine elevation stage was performed in alpine grasslands found above the timberline. Everywhere, agricultural or urban lands were avoided. At each site, about 10–12 soil cores of 10 cm diameter and 10–20 cm deep were collected until reaching a sufficient amount of soil (about 1 kg fresh weight) after the removal of big (>2 cm in diameter) rock particles. Soil samples were then placed in a cold room (4℃) within 24 hr after sampling; between one and four days later, from this well‐homogenized bulk soil material, a subsample of 100 g of fresh soil was used for extracting soil nematodes using the sieving and Baermann funnel method (Barker, 1985). The Baermann funnel method has been amply used for sampling nematodes across a wide variety of habitats and substrates, including soils and plant tissues (e.g., Freckman & Virginia, 1989; Kergunteuil, 2016; Son & Moon, 2013; Viglierchio & Schmitt, 1983).

All nematodes in each sample were then counted under an Olympus Stereo‐zoom SZX16 microscope, mounted into slides for identification to the genus level, and assigned to various functional guilds based on their trophic group and life history strategies (Yeates et al., 1993) (Table S3). Next, another subsample of the bulk soil was used for measuring soil parameters, including soil humidity, pH, conductivity, and temperature. For soil humidity, we calculated the difference between soil fresh weight and soil dry weight after 7 days at 70℃; pH and conductivity were measured using a pH meter/Conductometer (HANNA HI‐98129 pH, EC and TDS Meter, HANNA Instruments AG, Langnau bei Reiden, Switzerland), after mixing 50 g of this subsample with 100 ml of deionized water. Soil temperature was measured on site with a soil thermometer.

### 2.4 Nematode communities' functional characterization

Depending upon the abundance of functional guilds of nematodes, various indices were calculated so as to analyze the functional role of nematode‐based food webs along various mountain transects (Bongers & Bongers, 1998; Ferris et al., 2001). In order to do so, all identified nematodes were classified into five main trophic habits (bacterial‐feeders, fungal feeders, plant‐feeders, omnivores, and predators (Yeates et al., 1993)), and along the colonizer–persister (cp) scale (Bongers, 1990) (Table S3). Because we were working in yet largely unexplored territory (Northern India) in terms of nematode functional characterization, we resolved to only work with two major indices of relating nematodes' functional groups to ecosystem functioning: (a) the *Sigma maturity index* (ΣMI; Bongers, 1990), representing the proportions of the different cp groups for the whole nematode community, where higher values indicate that nematodes harboring “persister” life history traits are predominant within each of those different nematode categories. (b) The *Enrichment index* (EI; Ferris et al., 2001), which is based on the biomass of opportunistic nematodes that respond rapidly to the increase in bacterial and fungal populations that arise from organic matter decomposition. High values indicate high soil enrichment and high fertility. Biomass values were extrapolated from the NINJA (https://sieriebriennikov.shinyapps.io/ninja/).

### 2.5 Statistical analyses

All statistical analyses were performed using R software, version 4.0.3 (R Development Core Team, 2020).

1. Soil‐climate covariation: First, the effect of elevation on all individual soil and climate variables was tested using a mixed linear model (package *lme4* (Bates et al., 2015)) with “elevation” as fixed factor and “transect” as random factor. Second, we tested for a shared structure between soil properties and climatic conditions, which would represent a coupled soil–climate syndrome along elevation gradients, using a coinertia analysis. In other words, here we tested whether the matrices of soil parameters and climatic variables concomitantly vary across different sites. If this is the case, it would lead us to conclude that sites covary in their soil and climatic properties. The coinertia analyses were performed using the *ade4* package (Dray et al., 2003; Dray & Dufour, 2007), and the significance of the shared variance was assessed using a Monte Carlo test as implemented in *ade4*. When the coinertia analysis was significant (i.e., there is a significant soil/climate structuration across sites; see Figure S1), we performed a linear regression between combined soil–climate syndrome (coinertia 1) and elevation using a mixed linear model (package *lme4* (Bates et al., 2015)) with “elevation” as fixed factor and “transect” as random factor.
2. Nematodes trophic diversity covary with changes in soil and climatic conditions along the elevational gradient. First we assessed the effect of the coupled soil/climatic variables on the six major trophic groups of nematodes (herbivores, fungivores, bacterivores, predators, omnivores, and parasites) using a mixed linear model (package *lme4*) with “coinertia axis 1” as fixed factor and “transect” as random factor. Second, we scored the effect of individual soil and climatic variables on the different trophic groups by performing a distance‐based redundancy analysis (dbRDA) between nematode communities and (a) the climatic variable matrix and (b) the soil variable matrix. Distance matrices were built using Bray‐Curtis dissimilarity values, and significances were tested using permutational analyses of variance function *capscale*, the package *vegan* (Oksanen et al., 2013).
3. Nematode functional indices change along elevation. We assessed the effect of the coupled soil/climatic variables on the two nematode functional indices (ΣMI and EI) using a mixed linear model (package *lme4*) with “coinertia axis 1” as fixed factor and “transect” as random factor (Table 1).

**TABLE 1:** Type III Analysis of Variance Table with Satterthwaite's method for measuring the effect of the combined soil/climate variation (coinertia axis 1, Figure S1) on the different nematode trophic guilds

	SQ	NumDF	DenDF	*F*	Pr(>*F*)	
Herbivores	2,646.7	1	35.67	49.17	<0.001	***
Fungivores	221.8	1	37.77	20.93	<0.001	***
Bacterivores	29,906	1	35.54	129.46	<0.001	***
Predators	72.663	1	38	1.79	0.187	
Omnivores	13,012	1	35.67	84.63	<0.001	***
Parasites	2,650.3	1	20.71	6.43	0.019	*

Note

Signif. codes: 0 ‘***’ 0.001 ‘**’ 0.01 ‘*’ 0.05 ‘.’ 0.1 ‘ ’ 1.

## 3 RESULTS

**FIGURE 2:**
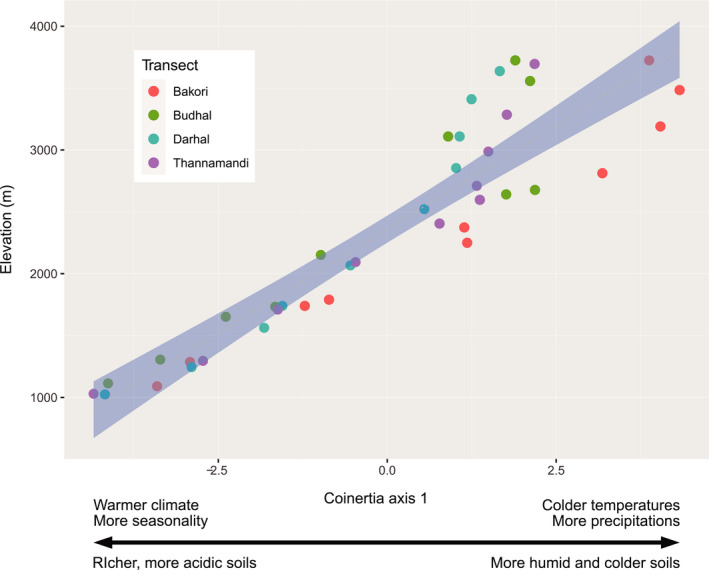
Climate and soil properties' covariation along elevation. Gray shading shows best fitting of the linear model with confidence intervals when the correlation is significant (*p* < .05). Dots are colored for distinguishing the four different elevation transects

**FIGURE 3:**
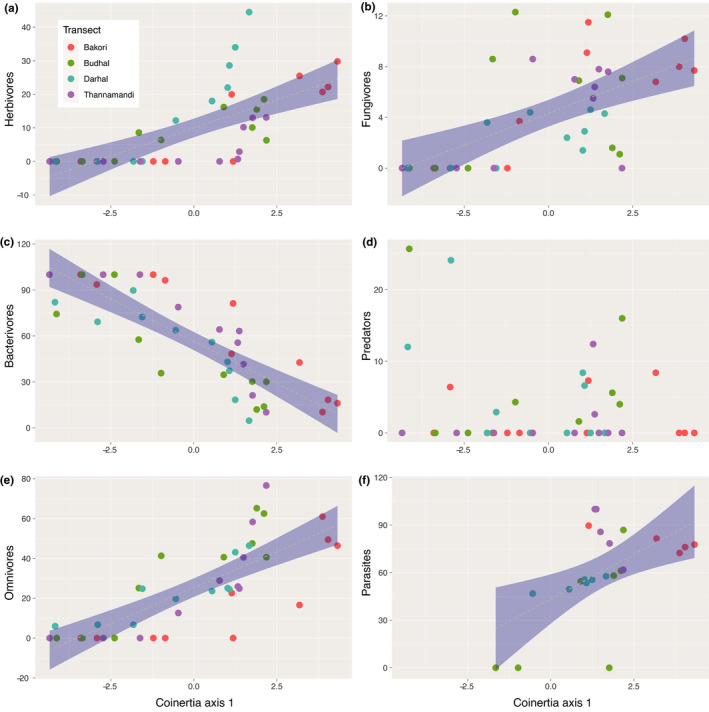
Effect of the coupled climate/soil variation along elevation and the abundance of different nematodes' trophic groups. (a) Herbivores, (b) fungivores, (c) bacterivores, (d) predators, (e) omnivores, (f) parasites. Abundances of nematodes represent densities per 100 g fresh soil. Different colors represent the sampling sites belonging to four mountain transects as shown in Table S1. Gray shading shows best fitting of the linear model with confidence intervals when the correlation is significant (*p* < .05). Dots are colored for distinguishing the four different elevation transects

**FIGURE 4:**
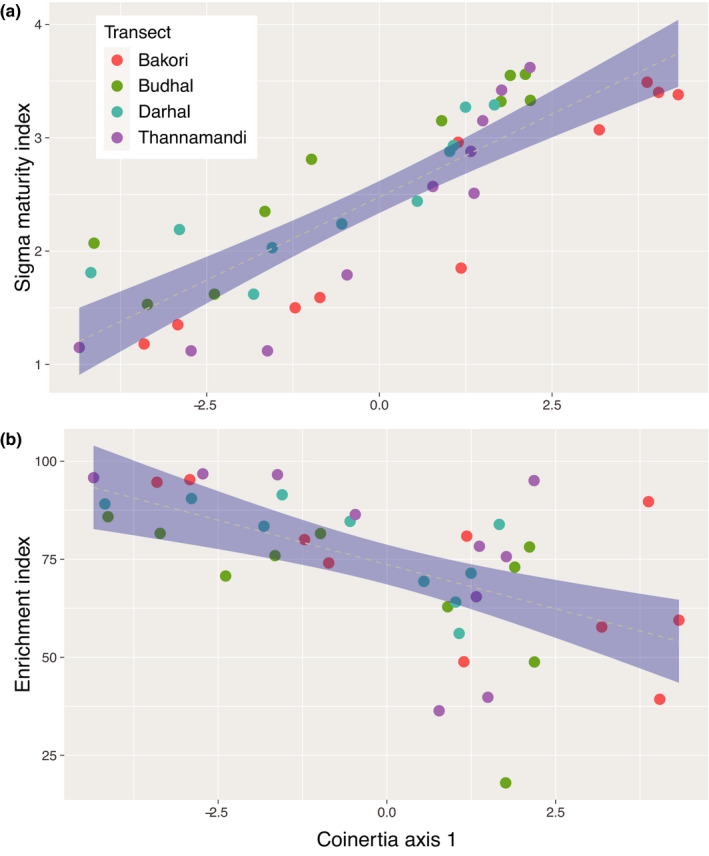
Effect of the coupled climate/soil variation along elevation and soil nematode functionality. Shown are linear model regression between the climate/soil coinertia axis 1 (see Figure S1), and (a) the Σ*MI*, (b) the *Enrichment index*. Gray shading shows best fitting of the linear model with confidence intervals when the correlation is significant (*p* < .05). Dots are colored for distinguishing the four different elevation transects

1. Soil‐climate covariation along elevation: We found that elevation was correlated with soil moisture, pH, temperature, and conductivity. Particularly, soil temperature (*t*_39_ = −31.70, *p*‐value <.001), conductivity (*t*_39_ = −11.34, *p*‐value <.001), and pH (*t*_39_ = −2.04, *p*‐value = .047) all decreased with elevation. From the lowest to the highest elevations, soil temperature decreased in average by 12.6 ± 0.4 degrees, conductivity decreased by a factor of 3.86 ± 1.14 mS/m, and pH decreased from 6.35 to 6.10. Soil moisture (*t*_39_ = 23.90, *p*‐value <.001) increased along the elevational gradient and was in the range of 25.69 ± 7.21. For climatic conditions, we found that high elevation sites were on average 9.25 ± 4.8 degrees colder, 16% more humid, and 10% less seasonal variation in precipitation than low‐elevation sites (Table S2). We also found that climatic and soil properties across sites were significantly correlated (Figure S1, Monte‐Carlo test based on 999 replicates, *r* = 0.71, simulated *p*‐value = .001). Finally, we found a positive correlation between the first axis of the soil/climate coinertia analysis (as a proxy of soil and climatic variables' correlation) and elevation (Figure 2, Type III ANOVA with Satterthwaite's method, *F*_1,35.01_, *p*‐value <.001).
2. Across 40 sites, we retrieved 15,091 nematodes per 100 g of soil, belonging to 47 genera (Table S3). Nematode communities observed along the elevational transects varied considerably in their composition (Figure S2). Low‐elevation soils found in forested areas, ranging from altitude 1,000 m to 2,500 m, were mainly composed of genera like *Plectus, Acrobeles, Mesorhabditis*, *Mylonchulus, Aphelenchus, Alaimus, Wilsonema,* and *Eudorylaimus*, while those found between the range of 2,500 m and 3,500 m were composed of genera like *Mesodorylaimus*, *Prodorylaimus*, *Aphelenchoides, Teratocephalus, Panagrolaimus, Tylencholaimus, Paratylenchus,* and *Helicotylenchus*. However, in the transition zones of elevation transects, various genera coexist, for example*, Helicotylenchus* and *Eudorylaimus*, which were found both in lower transects and in the middle transects, whereas genera like *Teratocephalus, Panagrolaimus,* and *Prodorylaimus* were found both in mid‐elevation transects and in the upper elevation transects at an altitude of 3,500 m a.s.l. Some genera were completely absent in some sites, but present in others. For example, the bacterivore genera *Cuticularia, Curviditis,* and *Rhabditis* were not found at high elevations, while *Longidorella, Nagelus* were not found at low elevations. Furthermore, above 3,500 m a.s.l., nematode communities mostly consisted of few herbivore and omnivore genera like *Pratylenchus, Longidorella, Prodorylaimus*, and *Panagrolaimus*. Overall, we observed a general increase in the number of nematodes along the elevational gradient, but the diversity of nematode communities declined at the highest elevations (above 3,500 m a.s.l.), which mostly consisted of herbivore and omnivore genera (Table 1, Figure 3). Among climatic variables, diurnal temperature range and isothermality were significantly associated with more bacterivores and predators, while seasonality was significantly correlated with more omnivores and herbivores (Figure S3, Table S4). Among soil variables, pH was negatively associated with fungivores, but positively associated with herbivores, while conductivity and temperature were positively associated with bacterivores, and finally, soil moisture was positively associated with omnivores (Figure S3, and Table S4).
3. We found that indices related to ecosystem properties varied with elevation (Figure S4), with nematode communities being associated with more productive and mature ecosystems found more likely living at low versus high elevation. Specifically, (a) the ΣMI showed an increase along the elevational gradient (Table 2, Figure 4a), depicting an increase in the relative abundance of persister nematodes when moving up along the elevational gradient, while (b) the EI showed a decline with elevation (Table 2, Figure 4b).

**TABLE 2:** Type III Analysis of Variance Table with Satterthwaite's method for (1) The Σ*MI*, (2) The *enrichment index*, (3) The *channel index*, and (4) The *metabolic footprints* (MF)

Indices	SQ	NumDF	DenDF	*F*	Pr(>*F*)	
Σ‐maturity	24.202	1	35.441	169.38	4.60E−15	***
Channel	2,854.3	1	37.365	9.2445	0.0043	**
Enrichment	4,548.9	1	37.727	18.323	0.0001231	***
MF	46,968	1	38	36.046	5.62E−07	***

Note

Signif. codes: 0 ‘***’ 0.001 ‘**’ 0.01 ‘*’ 0.05 ‘.’ 0.1 ‘ ’ 1.

## 4 DISCUSSION

Abiotic variation along elevational gradients shapes species diversity patterns, both for above‐ and belowground organisms, but the generalities of these patterns are still a matter of debate and vary across guilds of taxa (Sundqvist et al., 2013). Here, we studied elevational gradients in soil nematode functional structure in the lesser Himalayan range, and found that variation in nematode communities' functional composition along the elevational gradient was related to a shared structure of climatic and edaphic variables. Second, we found an increase in functional diversity and nematodes' footprints with elevation. Third, functional indices analyses highlighted a more stable ecological successional status and high amplitude of carbon utilization at high‐elevation sites, respectively. Below, we expand on these findings, and extrapolate on the relevance of soil nematodes' functional indices for characterizing ecosystem changes along ecological gradients.

### 4.1 Elevation effect on soil nematodes' trophic groups in relation to edaphic and climatic variables

We analyzed nematode communities' linkages to ecosystem function by studying sites‐specific soil and climatic variables along the elevational gradient. First, our results show an increase in soil moisture content along the elevation, which can be attributed to the increase in mean annual climatic precipitation and a decrease in precipitation seasonality. These properties were in turn related to increase in the abundance and diversity of nematodes along the elevation (Li et al., 2020). Indeed, we observed that most trophic groups of nematodes, except the bacterivores, which showed a decline, and the parasites, which showed no variation in abundance along elevation, all increased with elevation. Similarly, it was previously observed that soil nematodes' diversity is higher in cooler, more humid soils (Dong et al., 2017; Kergunteuil, 2016). Such trends might be counterintuitive, as the diversity and abundance of most taxonomic groups studied so far; e.g., plants (Bryant et al., 2008), arthropods (Hodkinson, 2005), or birds (Duclos et al., 2019; Patterson et al., 1998), all show either a steady decline or a bell‐shaped relationship with elevation (Brehm et al., 2007; Fernandez‐Conradi et al., 2020; Godschalx et al., 2019). This is thought to be the reflection of the climate becoming colder and harsher, and growing seasons becoming shorter at high elevation (Chapin & Korner, 1995), a pattern that we also observed: a decrease in mean diurnal temperature range, along with an increase in mean precipitation and a decrease in precipitation seasonality with the increasing elevation. Nematodes, in contrast, we observed to increase in abundance and functional (trophic) diversity with elevation. Other studies have shown the ability of nematodes to inhabit the harshest environments, such as the extreme polar regions (Loof, 1971; Yeates, 2010). Accordingly, we might speculate that nematodes can actually thrive more at high elevation, as they display adaptations to extreme low temperatures, such as the ability of supercooling and anhydrobiosis (Pickup, 1990; Pickup & Rothery, 1991; Wharton, 1995, 1996), while on the other hand, they might suffer from desiccation in warmer and drier conditions of low‐elevation sites (Procter, 1990).

Along with climatic variables, we also observed that soil conditions varied with elevation, and that they were also correlated with variation in nematodes' trophic groups. This is in line with previous results showing that soil variables are indeed important determinants of the composition of soil nematode communities (Li et al., 2020; Nielsen et al., 2014; Song et al., 2017; Wu et al., 2011). For instance, increased free water availability at high elevation is an important aspect for nematode movement and might thus be a contributing factor for promoting nematode populations in the alpine environment (Landesman et al., 2011). Taken together, we could argue that variation in nematode community composition and diversity along the elevation gradient could be explained by the shared effects of soil and climatic factors, highlighting the crucial role of interaction among multiple ecological factors on soil biodiversity. Nonetheless the climatic and edaphic factors explained only a proportion of the total variation in nematode diversity and composition (from 20% to 68%), suggesting that other potential and indirect explanatory variables, such as the vegetation characteristics or soil microbes, providing the habitat as well as food for nematodes, could also influence soil nematode diversity (Decaëns, 2010; Wardle, 2006). One caveat of our study is that we did not include classic diversity measures (e.g., Shannon, Simpson measures of entropy) of nematode communities along elevation gradients. The principal reason for this omission is that the taxonomic level at which we were working with, mostly using trophic and functional groups, does not allow a sufficient fine taxonomic resolution for these calculations to be any meaningful. Future work that scores soil nematode communities based on precise taxonomy, such as using DNA metabarcoding techniques, or through morphospecies scoring (Dell'Anno et al., 2015; Schenk et al., 2019), will very likely enable better estimates of alpha and beta diversity changes along elevation gradients.

### 4.2 Elevational variation in soil nematode functional indices related to ecosystem properties

While studies of taxonomic variation can inform on biodiversity changes along ecological gradients, the functional characterization of major players in the community, such as nematodes, is necessary to link biodiversity to ecosystem functioning (van den Hoogen et al., 2019, 2020; Tilman, 2001; Wall & Lynch, 2000). We here addressed the functional composition of nematodes by studying multiple integrative functional indices, including the ΣMI, which increased with elevation. Such patterns was likely driven by an increase in plant parasitic nematodes index (PPI) and stable soil conditions, that is, less disturbed environment (Bongers, 1990) at high elevation. We suspect that the reason for the observed increase in PPI with elevation goes hand in hand with changes in soil moisture, conductivity, and the climatic variables, particularly seasonality, precipitation, and temperature. Such conditions provide convenient environment for soil‐dwelling nematodes of larger body size, longer life cycles, that is, ‘persistent group’ of nematodes. That said, the increase in ΣMI along the elevational gradient depends on both the free‐living and the plant‐parasitic nematodes; however, high elevations were mostly inhabited by plant‐parasitic and omnivore nematode genera, likely due to their better tolerance to stress conditions (Bongers, 1990). A general decrease in the temperatures can also contribute to the maintenance of nematodes with long life cycles and low reproduction rates, thus, favoring the persister groups at high elevations. Furthermore, at high elevations, a denser root system provides a more suitable environment for herbivore and omnivore nematodes, by providing shelter from various abiotic stress, as well potentially providing enemy‐free zones (Kergunteuil, 2016).

Another index portraying the functional structure of nematode communities is the enrichment index (EI), which showed a negative correlation with elevation, although we detected strong variability right above the tree line (Figure 4b). Higher EI values indicate resource enrichment, and can be used to classify more undisturbed and more stable habitats (Ferris et al., 2001). Therefore, our findings suggest that across the elevational transects, soil resources are richer at lower than at higher altitudes. This might be explained by the slower erosion processes, and faster decomposition rates at lower elevations (Murphy et al., 1998), which lead to higher concentrations of nutrients in low‐elevation soils. Plant adaptations to high elevations include long leaf life span and slow growth. Moreover, high‐elevation soil organic matter decomposition is slow, likely due to thermal inhibition of the metabolic machinery (Pellissier & Rasmann, 2018). All together, these patterns result in decreased mineralization processes and decreased soil fertility (e.g., as shown by a decrease in soil conductivity at high elevation) (Wardle, 2006), and thus likely favoring enhanced fungal‐based energy flow at higher elevation (Wardle & Yeates, 1993; Zhao & Neher, 2014). Therefore, the EI can be used to extrapolate at which elevation the soil was sampled, as well as the quality of the soil in relation to productivity (Tsiafouli et al., 2017).

## 5 CONCLUSIONS

Changes in ecological factors like soil quality (Bongers, 1990), soil characteristics (de Goede & Bongers, 1994), habitat stability (Wasilewska, 1994), and climate (Crawford et al., 1991; Papatheodorou et al., 2004; Ruess et al., 1999; Sohlenius & Bostrom, 1999) strongly reflect on soil nematode functional composition, and therefore, it has been predicted that different ecosystems or habitats should sustain different communities of soil nematodes. Accordingly, we here showed that along elevation gradients, soil nematodes are particularly good bioindicators of local ecosystem properties (Overgaard, 1949; Procter, 1984; Yeates, 2003). Particularly, we showed that alpine ecosystems sustain a wider range of functional and taxonomic diversity than their respective low‐elevation sites. These results are in line with previous findings obtained along elevation gradients in the Alps (Kergunteuil, 2016), or across broad latitudinal gradients (van den Hoogen et al., 2019), and future work should focus on extending similar research to study the stability of such patterns across sites and years. Indeed, mountain ranges are unusually biodiverse, with copious accumulations of endemic species, which is the reflection of high variation in hydrology, in meteorology, as well as in ecological and evolutionary processes (Hoschitz & Kaufmann, 2004; Rahbek et al., 2019). Accordingly, mountains play an important role in sustaining Earth's biodiversity and ecosystem functioning (Körner, 2004). Soil nematodes contribute tremendously to such diversity, in terms of both taxonomic and functional diversity. Therefore, a better understating of the causes that generate nematodes biodiversity can inform on the impact of climate change and land‐use change on ecosystem functioning worldwide.

## CONFLICT OF INTEREST

None to declare.

## AUTHOR CONTRIBUTIONS

**Yasmeen Kouser:** Conceptualization (equal); data curation (equal); formal analysis (equal); funding acquisition (supporting); investigation (lead); methodology (lead); project administration (equal); resources (lead); software (equal); supervision (supporting); validation (equal); visualization (equal); writing‐original draft (equal); writing‐review & editing (equal). **Ali Asghar Shah:** Conceptualization (lead); data curation (equal); formal analysis (supporting); funding acquisition (equal); investigation (equal); methodology (equal); project administration (lead); resources (equal); software (equal); supervision (lead); validation (equal); visualization (equal); writing‐original draft (equal); writing‐review & editing (equal). **Sergio Rasmann:** Conceptualization (equal); data curation (equal); formal analysis (lead); funding acquisition (equal); investigation (equal); methodology (equal); project administration (equal); resources (equal); software (equal); supervision (lead); validation (equal); visualization (equal); writing‐original draft (lead); writing‐review & editing (lead).

## Supporting information

Supplementary Material

## Data Availability

Data underlying this article can be accessed on Dryad Digital Repository at https://doi.org/10.5061/dryad.v41ns1rx0, and used under the Creative Commons Attribution license.
