# Supplementary material for: The functional role and diversity of soil nematodes are stronger at high elevation in the lesser Himalayan Mountain ranges
Source: Ecol Evol. 2021 Sep 24;11(20):13793–804. doi: 10.1002/ece3.8061 (PMC8525141; doi:10.1002/ece3.8061)
Supplement: Supplementary file 1 — Supplementary Material [file ECE3-11-13793-s001.docx]

**Supplementary material for:**

**The functional role and diversity of soil nematodes are stronger at high elevation in the lesser Himalayan mountain ranges**

Yasmeen Kouser, Ali Asghar Shah, Sergio Rasmann

**Table S1.** Geographical details and soil properties (pH, Soil conductivity, soil relative humidity (moisture), and soil temperature) of the study sites across the four elevational transects in the Jammu and Kashmir region of India, where the nematode sampling was performed.

| Transect | Site | Latitude | Longitude | Elevation | pH | Conductivity | Moisture | Temperature |
| --- | --- | --- | --- | --- | --- | --- | --- | --- |
| Bakori | BK1 | 33.28539722 | 74.43906667 | 1091 | 6.84 | 4.00 | 16.10 | 19.20 |
| Bakori | BK2 | 33.31321667 | 74.46621389 | 1287 | 6.79 | 3.00 | 17.30 | 17.20 |
| Bakori | BK3 | 33.34730833 | 74.47665278 | 1740 | 6.19 | 3.00 | 21.00 | 15.50 |
| Bakori | BK4 | 33.36039722 | 74.48899167 | 1790 | 6.10 | 2.00 | 23.80 | 14.80 |
| Bakori | BK5 | 33.38785833 | 74.53160278 | 2250 | 6.50 | 2.00 | 25.10 | 13.50 |
| Bakori | BK6 | 33.41154722 | 74.51281389 | 2374 | 6.23 | 1.00 | 25.50 | 12.00 |
| Bakori | BK7 | 33.41745 | 74.52131111 | 2812 | 5.69 | 1.00 | 26.00 | 8.80 |
| Bakori | BK8 | 33.46079167 | 74.51494722 | 3190 | 6.39 | 0.50 | 28.20 | 7.20 |
| Bakori | BK9 | 33.46906389 | 74.548625 | 3484 | 6.07 | 0.50 | 28.00 | 5.00 |
| Bakori | BK10 | 33.48540556 | 74.54107222 | 3724 | 6.18 | 0.50 | 29.90 | 4.80 |
| Budhal | B1 | 33.27621667 | 74.56781389 | 1114 | 6.28 | 5.00 | 18.10 | 18.40 |
| Budhal | B2 | 33.28576389 | 74.60315833 | 1306 | 6.71 | 6.00 | 18.30 | 17.30 |
| Budhal | B3 | 33.32505833 | 74.53367778 | 1652 | 6.81 | 6.00 | 19.20 | 16.80 |
| Budhal | B4 | 33.35184444 | 74.52029722 | 1733 | 5.79 | 6.00 | 22.80 | 15.50 |
| Budhal | B5 | 33.38437778 | 74.63235 | 2152 | 5.21 | 5.00 | 24.10 | 14.20 |
| Budhal | B6 | 33.404975 | 74.66443611 | 2641 | 6.20 | 4.00 | 26.00 | 11.50 |
| Budhal | B7 | 33.410975 | 74.66950556 | 2677 | 6.26 | 3.00 | 27.00 | 9.30 |
| Budhal | B8 | 33.42700833 | 74.66713611 | 3109 | 6.39 | 3.00 | 29.00 | 7.10 |
| Budhal | B9 | 33.43686111 | 74.64867778 | 3557 | 6.04 | 0.50 | 31.00 | 5.00 |
| Budhal | B10 | 33.47514167 | 74.65258333 | 3724 | 6.22 | 0.10 | 32.20 | 4.90 |
| Darhal | D1 | 33.40183 | 74.32761 | 1025 | 6.71 | 6.00 | 17.10 | 17.20 |
| Darhal | D2 | 33.43499 | 74.34212 | 1245 | 6.70 | 6.00 | 18.30 | 16.90 |
| Darhal | D3 | 33.46251 | 74.35931 | 1563 | 6.61 | 5.00 | 20.00 | 15.00 |
| Darhal | D4 | 33.48139 | 74.38291 | 1740 | 6.70 | 4.00 | 24.80 | 15.70 |
| Darhal | D5 | 33.49889 | 74.39715 | 2067 | 6.20 | 4.00 | 25.10 | 13.10 |
| Darhal | D6 | 33.51884 | 74.43514 | 2522 | 6.21 | 3.00 | 27.00 | 11.00 |
| Darhal | D7 | 33.52808 | 74.45511 | 2854 | 6.28 | 3.00 | 27.00 | 9.80 |
| Darhal | D8 | 33.51179 | 74.48629 | 3109 | 6.31 | 3.00 | 29.20 | 7.20 |
| Darhal | D9 | 33.50299 | 74.49530 | 3410 | 6.06 | 1.00 | 30.00 | 6.00 |
| Darhal | D10 | 33.49419 | 74.51163 | 3637 | 6.20 | 0.50 | 31.20 | 5.10 |
| Thanamandi | TM1 | 33.4195 | 74.31674444 | 1030 | 5.71 | 7.00 | 16.50 | 16.20 |
| Thanamandi | TM2 | 33.49848889 | 74.35256944 | 1296 | 5.70 | 6.00 | 19.30 | 15.90 |
| Thanamandi | TM3 | 33.54326111 | 74.37642222 | 1710 | 6.51 | 4.00 | 20.10 | 15.00 |
| Thanamandi | TM4 | 33.55074167 | 74.40098333 | 2094 | 6.20 | 4.00 | 23.80 | 14.00 |
| Thanamandi | TM5 | 33.55978333 | 74.40981111 | 2405 | 6.28 | 4.00 | 26.10 | 12.10 |
| Thanamandi | TM6 | 33.56711667 | 74.40597222 | 2597 | 6.70 | 2.00 | 28.00 | 10.00 |
| Thanamandi | TM7 | 33.56357222 | 74.4216 | 2710 | 6.07 | 2.00 | 28.50 | 9.70 |
| Thanamandi | TM8 | 33.55550556 | 74.44253611 | 2987 | 6.41 | 1.00 | 29.70 | 8.20 |
| Thanamandi | TM9 | 33.54168889 | 74.44935833 | 3285 | 6.36 | 1.00 | 32.00 | 5.00 |
| Thanamandi | TM10 | 33.56174444 | 74.48722778 | 3695 | 5.90 | 0.50 | 32.90 | 4.10 |

**Table S2**. Climatic variables describing the climatic niche of the 40 sampling sites along 4 elevational transects. Bio1 = Mean Diurnal Range, Bio2 = Isothermality, Bio3 = Temperature Seasonality, Bio4 = Temperature Annual Range, Bio 5= Annual Precipitation, Bio6 = Precipitation of Wettest Month, Bio7 = Precipitation of Driest Month, Bio8 = Precipitation Seasonality, Bio9 = Precipitation of Warmest Quarter, Bio10 =Precipitation of Coldest Quarter.

| Transect | Site | Bio1 | Bio2 | Bio3 | Bio4 | Bio5 | Bio6 | Bio7 | Bio8 | Bio9 | Bio10 |
| --- | --- | --- | --- | --- | --- | --- | --- | --- | --- | --- | --- |
| Bakori | BK1 | 112 | 358 | 6723 | 313 | 1216 | 331 | 12 | 97 | 646 | 232 |
|  | BK2 | 112 | 358 | 6736 | 312 | 1222 | 325 | 13 | 94 | 639 | 240 |
|  | BK3 | 112 | 358 | 6829 | 312 | 1570 | 397 | 16 | 93 | 849 | 302 |
|  | BK4 | 112 | 358 | 6842 | 312 | 1531 | 353 | 16 | 82 | 758 | 318 |
|  | BK5 | 111 | 360 | 6881 | 310 | 1623 | 376 | 18 | 84 | 806 | 346 |
|  | BK6 | 111 | 360 | 6887 | 309 | 1552 | 362 | 18 | 85 | 780 | 324 |
|  | BK7 | 111 | 360 | 6970 | 308 | 1682 | 370 | 22 | 80 | 1106 | 374 |
|  | BK8 | 111 | 360 | 7035 | 308 | 1711 | 347 | 25 | 71 | 1035 | 394 |
|  | BK9 | 111 | 360 | 7102 | 308 | 1679 | 327 | 25 | 67 | 971 | 408 |
|  | BK10 | 111 | 360 | 7139 | 307 | 1471 | 273 | 23 | 63 | 793 | 367 |
| Budhal | B1 | 112 | 356 | 6780 | 315 | 1323 | 368 | 11 | 104 | 772 | 214 |
|  | B2 | 112 | 357 | 6793 | 314 | 1367 | 374 | 12 | 102 | 789 | 227 |
|  | B3 | 112 | 357 | 6837 | 313 | 1484 | 389 | 13 | 97 | 845 | 245 |
|  | B4 | 112 | 358 | 6844 | 312 | 1442 | 359 | 14 | 89 | 764 | 262 |
|  | B5 | 112 | 359 | 6893 | 311 | 1511 | 388 | 15 | 94 | 829 | 264 |
|  | B6 | 111 | 359 | 6995 | 311 | 1682 | 396 | 18 | 86 | 1184 | 321 |
|  | B7 | 111 | 359 | 6989 | 310 | 1683 | 378 | 19 | 82 | 1134 | 339 |
|  | B8 | 111 | 359 | 7069 | 310 | 1437 | 349 | 15 | 89 | 1040 | 237 |
|  | B9 | 111 | 360 | 7146 | 309 | 1451 | 319 | 16 | 78 | 941 | 276 |
|  | B10 | 111 | 360 | 7180 | 308 | 1375 | 314 | 15 | 82 | 934 | 234 |
| Darhal | D1 | 112 | 355 | 6802 | 315 | 1136 | 287 | 12 | 90 | 598 | 206 |
|  | D2 | 111 | 355 | 6822 | 314 | 1227 | 310 | 13 | 90 | 649 | 219 |
|  | D3 | 111 | 356 | 6862 | 313 | 1335 | 325 | 14 | 87 | 707 | 234 |
|  | D4 | 111 | 356 | 6883 | 312 | 1346 | 329 | 14 | 88 | 726 | 232 |
|  | D5 | 111 | 357 | 6925 | 311 | 1428 | 337 | 15 | 85 | 752 | 252 |
|  | D6 | 111 | 358 | 6987 | 310 | 1416 | 344 | 16 | 87 | 1018 | 245 |
|  | D7 | 111 | 358 | 7041 | 309 | 1435 | 333 | 16 | 84 | 996 | 238 |
|  | D8 | 111 | 359 | 7078 | 309 | 1365 | 327 | 15 | 84 | 962 | 230 |
|  | D9 | 111 | 359 | 7132 | 309 | 1373 | 324 | 15 | 84 | 957 | 228 |
|  | D10 | 111 | 360 | 7172 | 309 | 1371 | 315 | 15 | 82 | 938 | 233 |
| Thanamandi | TM1 | 112 | 354 | 6800 | 315 | 1092 | 266 | 12 | 86 | 566 | 206 |
|  | TM2 | 111 | 355 | 6827 | 313 | 1072 | 253 | 13 | 80 | 523 | 209 |
|  | TM3 | 111 | 356 | 6876 | 311 | 1184 | 284 | 14 | 82 | 587 | 225 |
|  | TM4 | 111 | 357 | 6936 | 310 | 1320 | 315 | 16 | 83 | 678 | 238 |
|  | TM5 | 111 | 357 | 6979 | 310 | 1431 | 325 | 18 | 79 | 961 | 253 |
|  | TM6 | 111 | 357 | 7011 | 310 | 1470 | 320 | 20 | 74 | 946 | 272 |
|  | TM7 | 111 | 358 | 7025 | 310 | 1475 | 328 | 18 | 77 | 970 | 259 |
|  | TM8 | 111 | 358 | 7071 | 309 | 1435 | 310 | 17 | 74 | 914 | 243 |
|  | TM9 | 111 | 358 | 7123 | 309 | 1477 | 319 | 17 | 75 | 951 | 248 |
|  | TM10 | 111 | 359 | 7192 | 308 | 1366 | 286 | 17 | 72 | 850 | 240 |

**Table S3.** Functional classification of soil nematodes found along 40 sites in the Jammu and Kasmir region.

| **Coloniser-persister classification of nematodes, feeding type and mass(ug)** | | | | |
| --- | --- | --- | --- | --- |
| **Genera** | **C-p class** | **P-p class** | **Feeding type** | **Mass (mg)** |
| *Dorylaimellus* | 0 | 5 | Herbivores - sedentary parasites | 0.303 |
| *Helicotylenchus* | 0 | 3 | Herbivores - semi-endoparasites | 0.287 |
| *Longidorella* | 0 | 4 | Herbivores - ectoparasites | 0.497 |
| *Nagelus* | 0 | 3 | Herbivores - ectoparasites | 0.67 |
| *Paratylenchus* | 0 | 2 | Herbivores - ectoparasites | 0.053 |
| *Pratylenchus* | 0 | 3 | Herbivores - migratory endoparasites | 0.126 |
| *Rotylenchus* | 0 | 3 | Herbivores - semi-endoparasites | 3.559 |
| *Tylenchorhynchus* | 0 | 3 | Herbivores - ectoparasites | 0.234 |
| *Aphelenchoides* | 2 | 0 | Fungivores | 0.145 |
| *Aphelenchus* | 2 | 0 | Fungivores | 0.231 |
| *Deladenus* | 2 | 0 | Fungivores | 1.095 |
| *Diphtherophora* | 3 | 0 | Fungivores | 0.504 |
| *Tylencholaimellus* | 4 | 0 | Fungivores | 0.564 |
| *Tylencholaimus* | 4 | 0 | Fungivores | 0.472 |
| *Acrobeles* | 2 | 0 | Bacterivores | 0.6 |
| *Alaimus* | 4 | 0 | Bacterivores | 0.534 |
| *Ceratoplectus* | 2 | 0 | Bacterivores | 0.462 |
| *Curviditis* | 1 | 0 | Bacterivores | 3.788 |
| *Cuticularia* | 1 | 0 | Bacterivores | 1.172 |
| *Diploscapter* | 1 | 0 | Bacterivores | 0.261 |
| *Eucephalobus* | 2 | 0 | Bacterivores | 0.243 |
| *Heterocephalobus* | 2 | 0 | Bacterivores | 0.356 |
| *Mesorhabditis* | 1 | 0 | Bacterivores | 0.568 |
| *Monhystera* | 2 | 0 | Bacterivores | 1.185 |
| *Panagrolaimus* | 1 | 0 | Bacterivores | 0.612 |
| *Pelodera* | 1 | 0 | Bacterivores | 7.856 |
| *Plectus* | 2 | 0 | Bacterivores | 0.902 |
| *Prismatolaimus* | 3 | 0 | Bacterivores | 0.413 |
| *Protorhabditis* | 1 | 0 | Bacterivores | 0.275 |
| *Rhabditis* | 1 | 0 | Bacterivores | 7.5 |
| *Teratocephalus* | 3 | 0 | Bacterivores | 0.078 |
| *Wilsonema* | 2 | 0 | Bacterivores | 0.054 |
| *Clarkus* | 4 | 0 | Predators | 4.389 |
| *Discolaimus* | 5 | 0 | Predators | 2.928 |
| *Enchodelus* | 4 | 0 | Predators | 3.434 |
| *Mononchus* | 4 | 0 | Predators | 3.868 |
| *Mylonchulus* | 4 | 0 | Predators | 1.745 |
| *Paravulvus* | 5 | 0 | Predators | 1.23 |
| *Tripyla* | 3 | 0 | Predators | 5.02 |
| *Allodorylaimus* | 4 | 0 | Omnivores | 3.824 |
| *Dorylaimus* | 4 | 0 | Omnivores | 39.281 |
| *Epidorylaimus* | 4 | 0 | Omnivores | 1.647 |
| *Eudorylaimus* | 4 | 0 | Omnivores | 3.09 |
| *Heterodorus* | 4 | 0 | Omnivores | 3.075 |
| *Mesodorylaimus* | 4 | 0 | Omnivores | 1.306 |
| *Prodorylaimus* | 4 | 0 | Omnivores | 5.787 |
| *Thornia* | 4 | 0 | Omnivores | 0.875 |

**Table S4**. Distance-based redundancy analysis (dbRDA) results for correlating soil nematode community trophic structure with either climatic or edaphic (soil) variables. Shown are both the significance of the effects on individual variables and along the PCA axes of the ordination.

|  | Variable | Df | SSQ | F | Pr(>F) |  | PCA | Df | SSP | F | Pr(>F) |  |
| --- | --- | --- | --- | --- | --- | --- | --- | --- | --- | --- | --- | --- |
| Climate | Diurnal range | 1 | 0.69 | 3.84 | 0.00 | ** | CAP1 | 1 | 1.65 | 9.13 | 0.00 | *** |
|  | Isothermality | 1 | 0.84 | 4.69 | 0.00 | *** | CAP2 | 1 | 0.43 | 2.36 | 0.01 | ** |
|  | Temperature seasonality | 1 | 0.53 | 2.92 | 0.00 | ** | CAP3 | 1 | 0.25 | 1.38 | 0.73 |  |
|  | Temperature range | 1 | 0.19 | 1.07 | 0.29 |  | CAP4 | 1 | 0.20 | 1.10 | 1.00 |  |
|  | Annual precipitation | 1 | 0.27 | 1.49 | 0.09 | . | CAP5 | 1 | 0.17 | 0.95 | 1.00 |  |
|  | Precipitation wettest month | 1 | 0.16 | 0.90 | 0.54 |  | CAP6 | 1 | 0.16 | 0.89 | 1.00 |  |
|  | Precipitation driest month | 1 | 0.14 | 0.78 | 0.82 |  | CAP7 | 1 | 0.16 | 0.87 | 1.00 |  |
|  | Precipitation seasonality | 1 | 0.23 | 1.26 | 0.13 |  | CAP8 | 1 | 0.16 | 0.86 | 1.00 |  |
|  | Precipitation warmest quarter | 1 | 0.18 | 0.97 | 0.39 |  | CAP9 | 1 | 0.13 | 0.74 | 1.00 |  |
|  | Precipitation coldest quarter | 1 | 0.17 | 0.94 | 0.44 |  | CAP10 | 1 | 0.11 | 0.59 | 0.99 |  |
|  | Residual | 29 | 5.22 |  |  |  | Residual | 29 | 5.22 |  |  |  |
| Soil | pH | 1 | 0.41 | 2.18 | 0.01 | ** | CAP1 | 1 | 1.60 | 8.62 | 0.00 | *** |
|  | Conductivity | 1 | 0.97 | 5.22 | 0.00 | *** | CAP2 | 1 | 0.20 | 1.06 | 0.82 |  |
|  | Moisture | 1 | 0.46 | 2.48 | 0.00 | ** | CAP3 | 1 | 0.17 | 0.93 | 0.94 |  |
|  | Temperature | 1 | 0.28 | 1.50 | 0.07 | . | CAP4 | 1 | 0.14 | 0.78 | 0.97 |  |
|  | Residual | 35 | 6.51 |  |  |  | Residual | 35 | 6.51 |  |  |  |

Signif. codes: 0 ‘***’ 0.001 ‘**’ 0.01 ‘*’ 0.05 ‘.’ 0.1 ‘ ’ 1

**Figure S1.** Coinertia analysis between cliamtic and edaphic (soil) properites. The corrlation between the two matrices is significant (Monte-Carlo test based on 999 permutations: r = 0.71, simulated p-value: 0.001).

**Figure S2**. Abundance of nematodes across elevation gradients. Shown are the densities of major nematode groups (per 100 g of soil) in the subalpine (between 1000 and 2500 m above sea level) and the alpine zone (> 2500 m above sea level).

**Figure S3**. Distance-based redundancy analysis (dbRDA) results to detect linear relationship between the nematode communities’ trophic structure (in red) and A) the climatic niche of each site, and B) the soil properties of each site. Distance matrices built using Bray-Curtis dissimilarity values, and significances were tested using permutational analyses of variance.


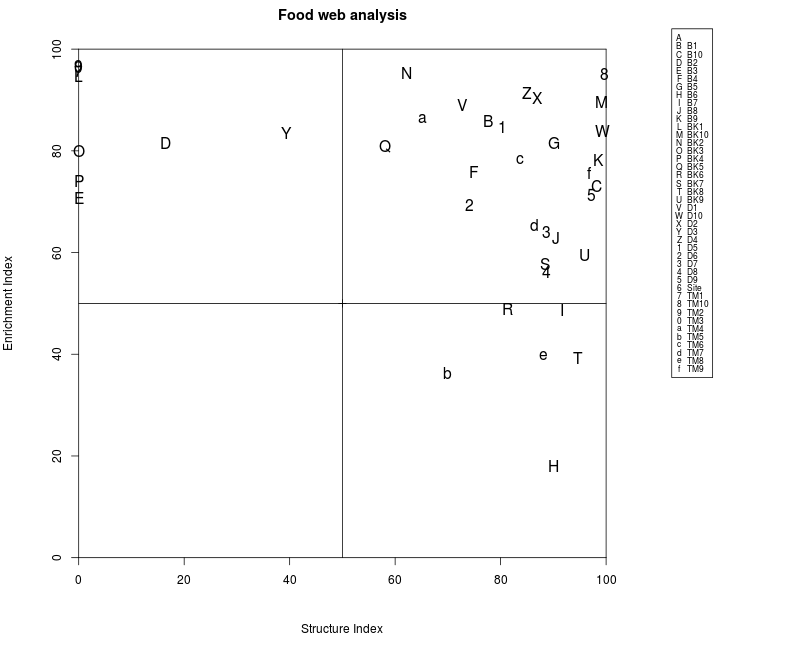


**Figure S4**. Faunal profile of the nematode communities sampled. Letters and numbers correspond to the different sites along the four elevational transects and the ten elevations per transect. B = Budhal, BK = Bakori, D = Darhal, TM = Thannamandi.
